# Trends in cocirculation of oncogenic HPV genotypes in single and multiple infections among the unvaccinated community

**DOI:** 10.1002/jmv.27706

**Published:** 2022-03-24

**Authors:** Manijheh Vazifehdoost, Fatemeh Eskandari, Amir Sohrabi

**Affiliations:** ^1^ Reference Health Laboratory Ministry of Health and Medical Education Tehran Iran; ^2^ Department of Biology, East Tehran Branch Islamic Azad University Tehran Iran; ^3^ Saeed Pathobiology Laboratory Tehran Iran; ^4^ Department of Medical Epidemiology and Biostatistics Karolinska Institutet Stockholm Sweden

**Keywords:** genital cancer, genotyping, HPV, Iran, multiple infections, sequencing

## Abstract

Cocirculation of multiple human papillomavirus (HPV) infections with low, probably high, and high‐risk genotypes are to be associated with various grades of infections and cancer progression. The oncogenic high‐risk HPVs are distributed and cocirculated throughout the world. This study was investigated to identify HPV genotypes related to genital disorders in unvaccinated women. The subjects were referred from clinics to a molecular lab for HPV testing in Iran as a low‐coverage vaccinated country. HPVs DNAs of cervical scrapping and genital tissue specimens of 1,133 un‐vaccinated women were genotyped using an in vitro diagnostic line probe (reverse hybridization) assay. In addition, phylogenetic trees were constructed on 100 MY09/MY11 polymerase chain reaction (PCR) amplicons of common genotypes of HPV L1 gene by Sanger sequencing. The mean age of the population study was 32.7 ± 8.0 and the mean age of HPV‐positive cases was 31.6 ± 7.8. HPV DNA was detected in 57.8% (655/1133) of women subjects and 42.2% (478/1133) of cases were undetected. Among 655 HPV‐positive cases, 639 subjects (56.4%) were related to defined genotypes and 16 subjects (1.4%) were untypeable. The highest prevalence rate of HPV genotypes was identified in the 25–34 years. The top 6 dominant HPVs in single and multiple genotypes were HPV6 (284/655 [43.4%]), HPV16 (111/655 [16.9%]), HPV31 (72/655 [11%]), HPV53 (67/655 [10.2%]), HPV11 (62/655 [9.5%]), and HPV52 (62/655 [9.5%]). Moreover, single, multiple and untypeable HPV genotypes were diagnosed as follows: 1 type (318/655 [48.5%]), 2 types (162/655 [24.8%]), 3 types (83/655 [12.7%]), 4 types (42/655 [6.5%]), more than 5 types (34/655 [5.3%]), and 1.4% un‐typeable subjects. The sequenced partial L1 gene of HPV genotypes (GenBank databases under the accession numbers: MH253467‐MH253566) confirmed and determined the cocirculated HPV genotypes' origins and addressed helpful insights into the future viral epidemiology investigations. Multiple HPV infections and cocirculation of various oncogenic HPV genotypes among the normal population (women and men) with asymptomatic forms are still challenging in unvaccinated communities. The preventive and organized surveillance programs for HPV screening are needed to be considered and compiled by health policy makers of low or unvaccinated countries.

## 1 INTRODUCTION

Human papillomavirus (HPVs) infections are responsible for several genital, anogenital, oropharyngeal, head–neck cancers, and sexually transmitted infections. The *Papillomaviridae* family is a nonenveloped, double‐stranded DNA with genomes around 5.7–8.7 kbp. E6 and E7 proteins are the most important genome regions to induce cancer progression. HPVs classification by the International Committee of Taxonomy Viruses (ICTV) is based on pairwise nucleotide sequence identity across the L1 (major capsid protein) open reading frame. The long control genome (LCR) does not encode any genes, and therefore is able to accumulate and tolerate more mutations.^1^, ^2^, ^3^, ^4^, ^5^ HPV is categorized into various lineages and sublineages that are differentiated in viral persistence and progression from mild infections to precancerous/cancer. The taxonomy database shows HPV isolates are classified into five genera: α‐papillomavirus, β‐papillomavirus, γ‐papillomavirus, mu‐papillomavirus, and nu‐papillomavirus.^1^, ^2^, ^5^, ^6^, ^7^ HPV genotypes are classified into oncogenic high‐risk HPVs (HR‐HPVs), nononcogenic low‐risk HPVs (LR‐HPVs), and probably high‐risk (pHR) HPV genotypes. HR‐HPVs are a major cause of genital malignancies, cervical intraepithelial neoplasia lesions, and cervical cancer that are increasing in low and un‐HPV vaccinated countries. Specific genomic modifications of nucleotide sequences in HR‐HPV genotypes can increase the formation of asymptomatic and noninvasive forms to invasive symptoms and cancer. It might also affect the pathogenicity of LR‐HPVs to induce severe symptoms and invasive genital malignancies. The environmental transmissions of HPV infections play a major role in distributing the genital and anogenital symptoms mainly genital warts.^8^, ^9^, ^10^, ^11^ Some scientific documents presented are about uncommon environmental routes of spreading HPV genotypes among the general population. For instance, from beauty clinics by accessories of body waxing, tattoo, usage of share towels in the swimming pool and sauna, sonography probes, and so forth.^12^, ^13^, ^14^, ^15^, ^16^, ^17^, ^18^, ^19^, ^20^, ^21^ Intervention measures based on Pap smear screening and HPV testing of the general population with asymptomatic HPV form lead to prevent mild infections and genital lesions to precancerous and cervical cancer. Currently, the HPV vaccine is the most efficient and critical prophylactic strategy in cervical cancer related to HPV genotypes. The tetravalent and nonavalent vaccines are Gardasil® (HPVs 16, 18, 6, and 11), Gardasil 9® (HPVs 16, 18, 31, 33, 45, 52, 68, 6, and 11), respectively. In addition, Cervarix ® vaccine (HPVs 16 and 18) has also been used in the past decade. Moreover, the poor HPV diagnosis and genotyping using unapproved assays and unorganized screening programs are still challenging in low‐income and developing countries.^22^, ^23^, ^24^ HPVs detection based on the L1 capsid gene region might not detect all HPVs and fully estimate HPV disorders burden. Sequence analysis of the L1 is considered as the choice method for types’ discrimination, lineages, and sublineages to confirm the consequences. Multiple HPV infections are frequently reported among women and men suffering from sexually transmitted infections. It is continuously discussed the genotypes are randomly distributed or there are interactions between HPV types whether there are trends in circulation of specific genotypes. The synergistic interactions across multiple HPV genotypes might affect cervical malignancies' progression and risk of invasive genital infections.^9^, ^25^, ^26^, ^27^, ^28^ However, HPV molecular epidemiological studies support for better understanding of trends in cocirculation of HR and LR dominant genotypes throughout the world. The findings especially HPV diagnosis at an early stage of disorders can be greatly prevented cancers in low and unvaccinated countries. In this study, we performed HPV genotyping on genital scrapping and tissues women specimens. The subjects were referred to a molecular lab for HPV testing in Iran. In addition, certain positive HPV samples were sequenced (based on the quality of polymerase chain reaction [PCR] products and HPV genotypes detected) for determination of cocirculated HPV genotypes origins in single and multiple infections.

## 2 MATERIALS AND METHODS

### 2.1 population subjects

The 1133 archival cervical scrapping and genital tissue specimens collected from unvaccinated women were referred to a private pathobiology laboratory, Tehran, Iran, from April 2012 to October 2015. The specimens were included vaginal brush 789/1133 (69.6%), vaginal brush with genital wart 248/1133 (21.8%), genital wart 83/1133 (7.3%), formalin‐fixed embedded tissue 4/1133 (0.4%), genital wart with anal wart 3/1133 (0.3%), anal wart 2/1133 (0.2%), vaginal brush with anal wart 2/1133 (0.2%), vaginal brush with anal brush 1/1133 (0.1%), and vaginal brush with genital/anal wart 1/1133 (0.1%). The mean age of the population study was 32.7 ± 8.0 and the mean age of HPV‐positive cases was 31.6 ± 7.8. The clinical data were recorded from medical documents. The cross‐sectional descriptive study was conducted according to the ethical guidelines of the Declaration of Helsinki, and approved by the deputy research of East Tehran Branch, Islamic Azad University, Tehran (No. 28330503961002). The liquid‐based cytology (LBCs) and tissue samples were stored at −20°C until experiments.

### 2.2 HPV testing

DNA of HPV genotypes was extracted from LBCs and genital tissues using the High Pure PCR Template Preparation Kit (Roche^©^), according to the instruction manual. HPV genotyping was performed by an approved CE marked and In Vitro Diagnostic (IVD) Kit including INNO‐LiPA® HPV Genotyping Extra I and II (Fujirebio Diagnostics Inc.). The kit was able to detect 18 HR types (HPVs 16, 18, 26, 31, 33,35, 39, 45, 51, 52, 56, 58, 59, 66, 68, 70, 73, and 82), 9 LR types (HPVs 6, 11, 40, 42, 43, 44, 54, 61, and 81), 1 probably HR type (HPV53), and 5 non HR–LR genotypes (HPVs 62, 67, 69/71, 83, and 89).^24^, ^29^

### 2.3 Phylogenetic analysis

The HPV‐positive PCR products were amplified using MY09/MY11 primers (3). The positive amplicon around 450 bp was considered HPV‐positive underwent electrophoresis on a 1.5% agarose gel. The gel was stained by ethidium bromide and was visualized under ultraviolet light. An approximately 15 µl of amplicon reactions were sent to Pooya Gostar Gene© for Sanger sequencing by a BigDye Terminator Cycle Sequencing Kit on an ABI Prism 3730/3100 DNA analyzer (Applied Biosystems Inc.). The FASTA files of sequences were trimmed and aligned using BioEdit (version 7.2) and ClustalW software. The phylogenetic analysis was constructed using the MEGA software package (version 6) on multiple alignment outputs that were also performed basic local alignment search tool (BLAST) using NCBI (https://blast.ncbi.nlm.nih.gov/Blast.cgi) by the neighbor‐joining method with 1000 bootstrap repetitions to find any similarities and to determine the HPV genotypes origins compared to HPV reference isolates.

## 3 RESULTS

**Figure 1:**
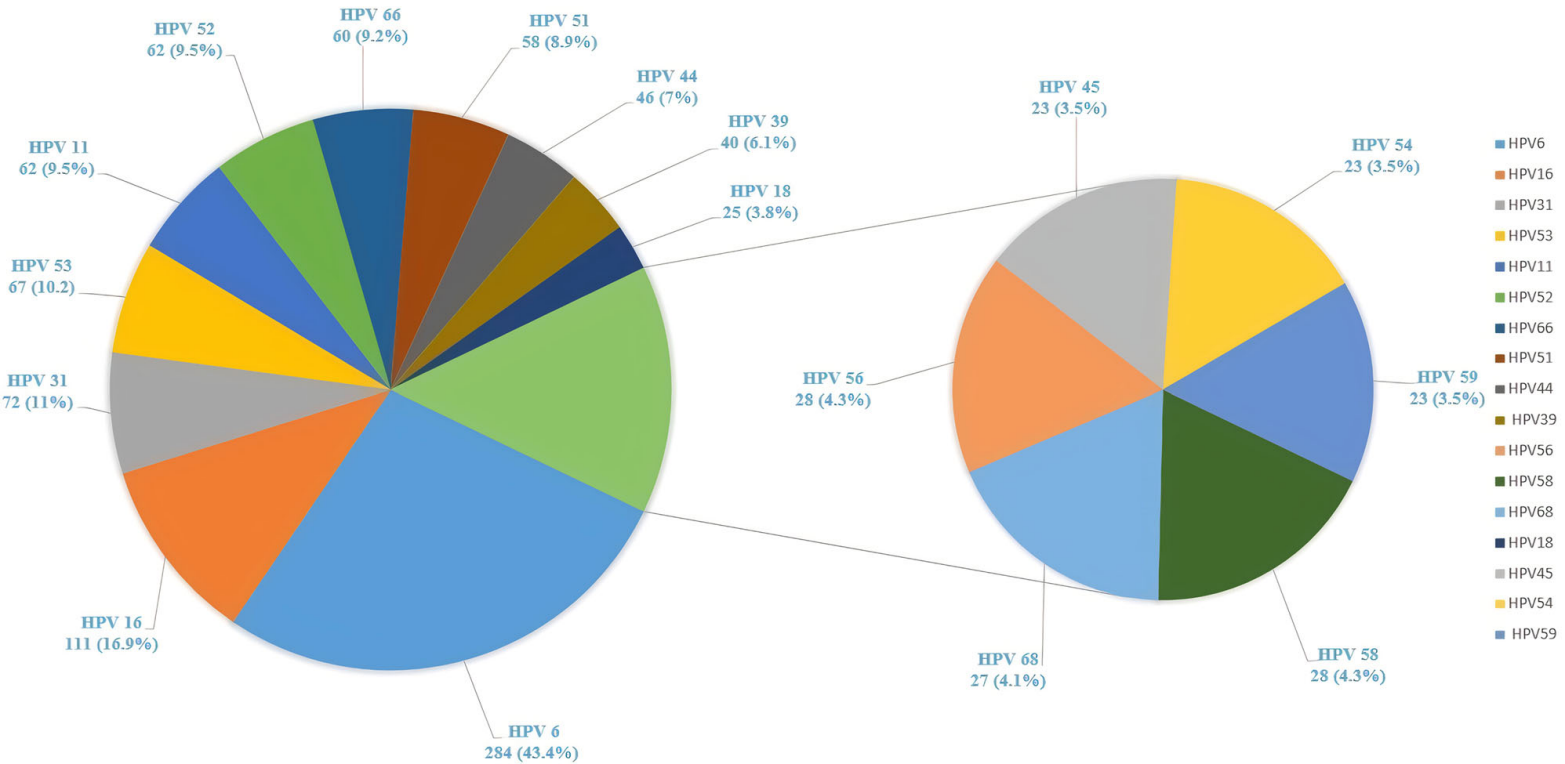
The highest prevalence rate of HPV genotypes detected in single and multiple infections. HPV, human papillomavirus

**Figure 2:**
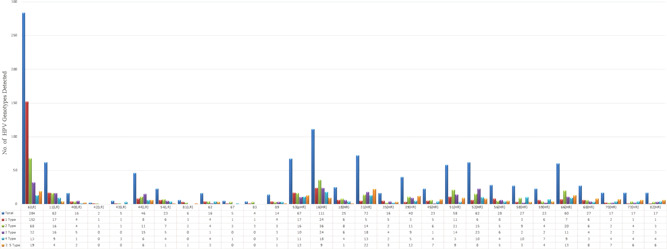
Frequency of single and multiple HPV genotypes detected. HPV, human papillomavirus; HR, high risk; LR, low risk; pHR, probably high risk

**Figure 3:**
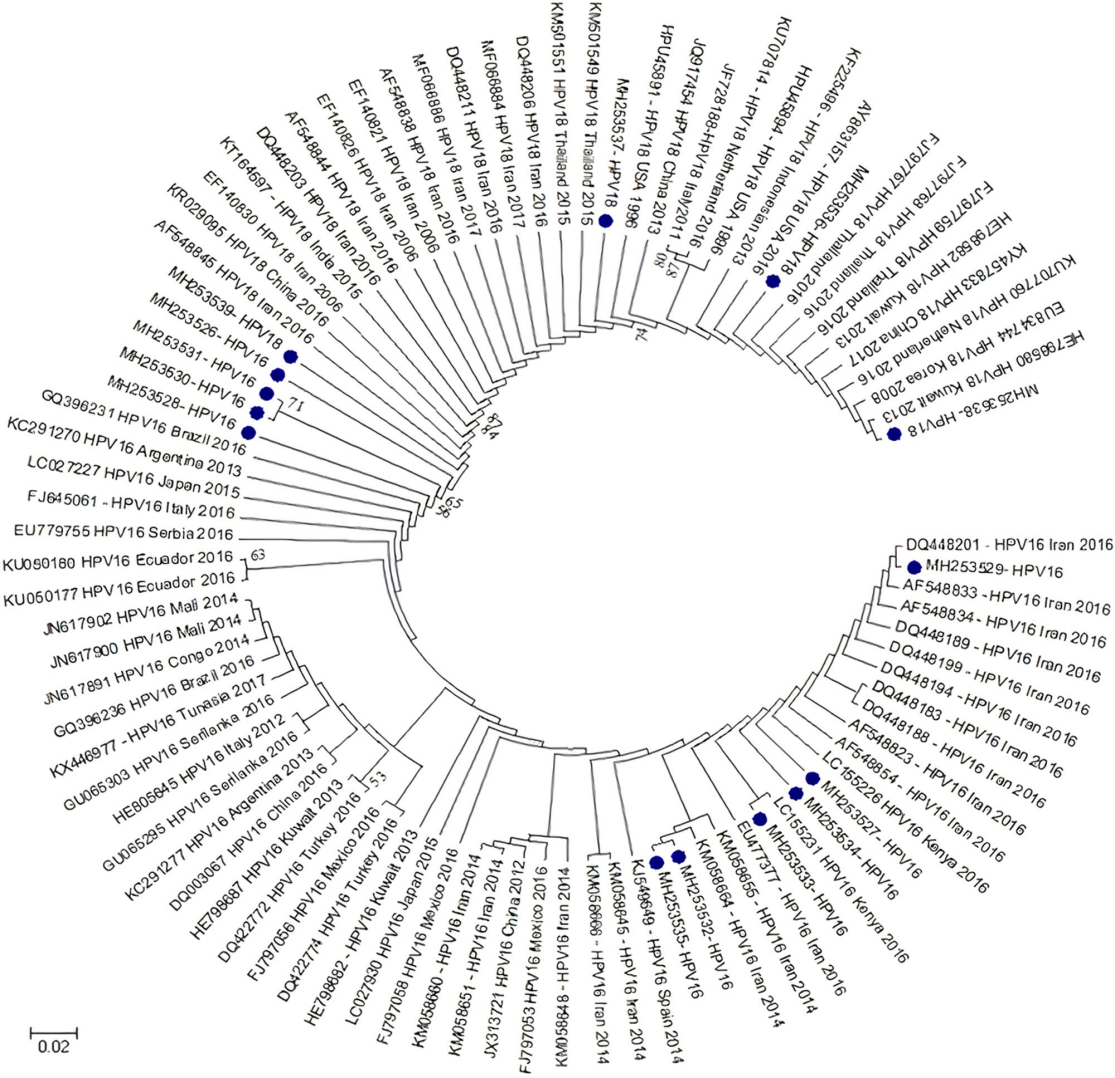
Phylogenetic tree constructed from the L1 (major capsid protein) nucleotide sequence of the isolated high‐risk HPVs 16 and 18 genotypes from Iran and reference HPV genotypes. The tree was elaborated by the neighbor‐joining method using MEGA 6. The numbers adjacent to the nodes represent the percentage of bootstrap support (of 1000 replicates) for each node. Bootstrap values lower than 50% are not shown. The scale bar corresponds to 0.02 substitutions/site. HPVs 16 and 18 analyzed in the present study are shown by blue circles. HPV, human papillomavirus

**Figure 4:**
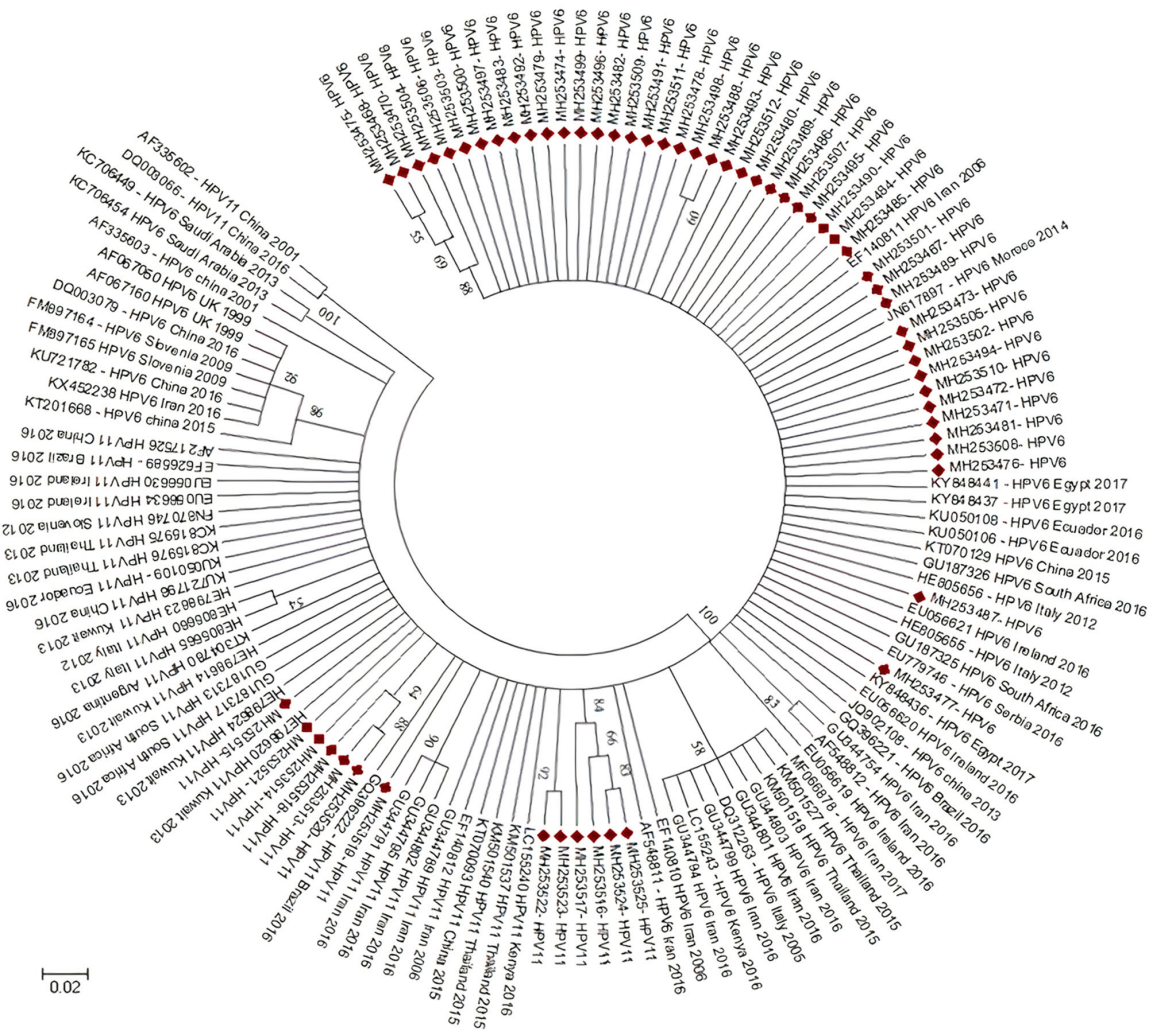
Phylogenetic tree constructed from the L1 (major capsid protein) nucleotide sequence of the isolated low‐risk HPVs 6 and 11 genotypes from Iran and reference HPV genotypes. The tree was elaborated by the neighbor‐joining method using MEGA 6. The numbers adjacent to the nodes represent the percentage of bootstrap support (of 1000 replicates) for each node. Bootstrap values lower than 50% are not shown. The scale bar corresponds to 0.02 substitutions/site. HPVs 6 and 11 analyzed in the present study are shown by red cubics. HPV, human papillomavirus

**Figure 5:**
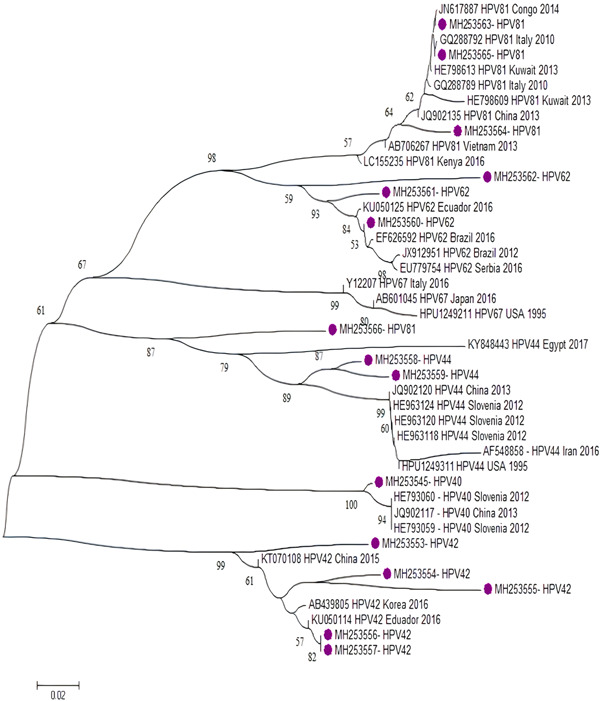
Phylogenetic tree constructed from the L1 (major capsid protein) nucleotide sequence of the isolated low‐risk HPV genotypes from Iran and reference HPV genotypes. The tree was elaborated by the neighbor‐joining method using MEGA 6. The numbers adjacent to the nodes represent the percentage of bootstrap support (of 1000 replicates) for each node. Bootstrap values lower than 50% are not shown. The scale bar corresponds to 0.02 substitutions/site. Low‐risk HPV genotypes analyzed in the present study are shown by purple circles. HPV, human papillomavirus

**Figure 6:**
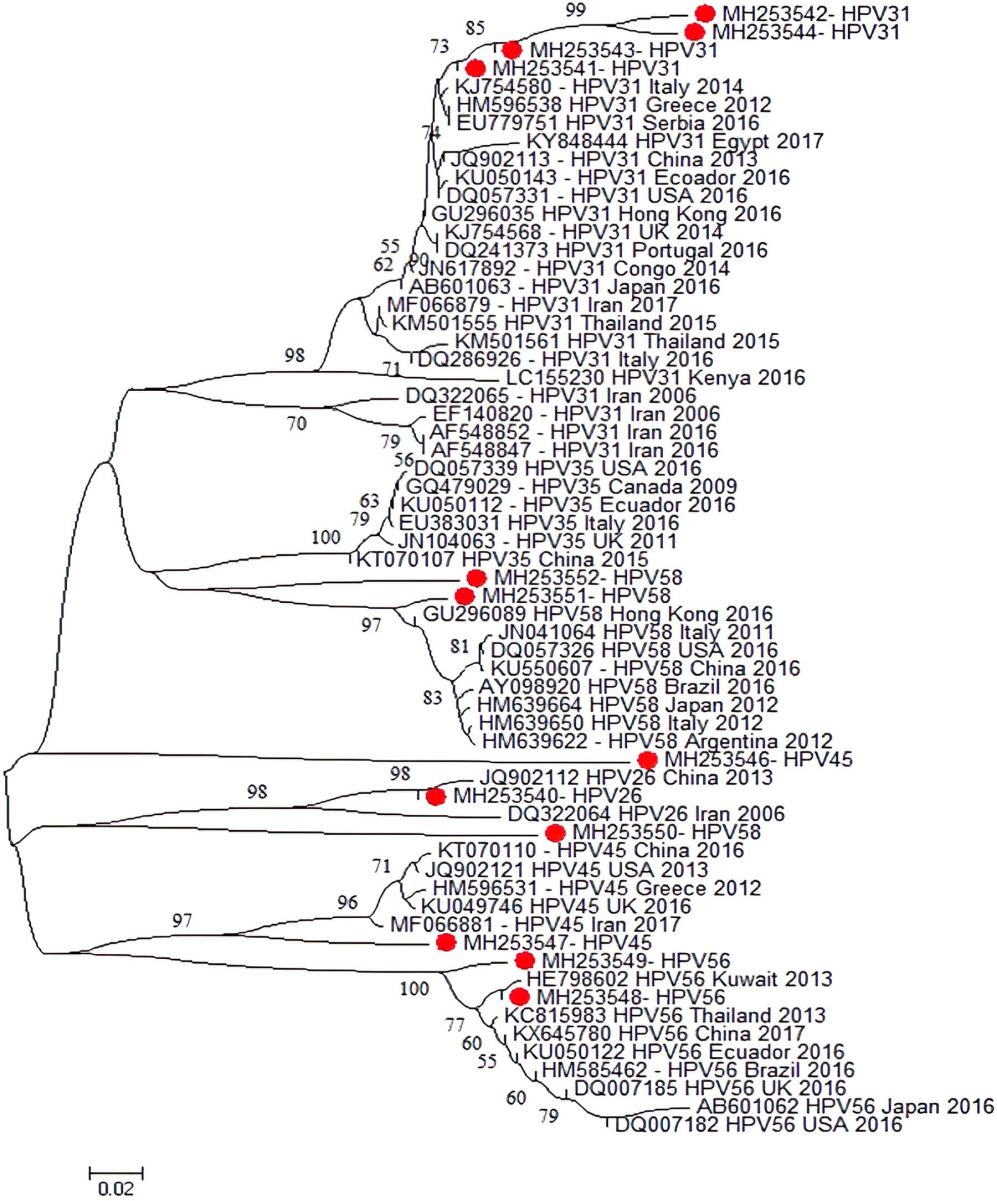
Phylogenetic tree constructed from the L1 (major capsid protein) nucleotide sequence of the isolated high‐risk HPV genotypes from Iran and reference HPV genotypes. The tree was elaborated by the neighbor‐joining method using MEGA 6. The numbers adjacent to the nodes represent the percentage of bootstrap support (of 1000 replicates) for each node. Bootstrap values lower than 50% are not shown. The scale bar corresponds to 0.02 substitutions/site. High‐risk HPV genotypes analyzed in the present study are shown by red circles. HPV, human papillomavirus

HPV genotypes were detected in 655 (57.8%) samples of 1,133 subjects and 478 (42.2%) cases were HPV negative. Among 655 HPV‐positive cases, 639 subjects (56.4%) were related to defined genotypes and 16 subjects (1.4%) were un‐typeable. The un‐typeable cases had positive signals on INNO‐LiPA® and PCR MY09/MY11 amplification methods although there were not associated with specific genotypes. The highest prevalence rate of HPV genotypes was observed in the 25–34 years of age group (Table 1). The highest prevalence rate of HPV genotypes detected in single and multiple infections in 655 positive cases are shown in Figure 1. The prevalence rate of the rest HPV diagnosed genotypes was less than 3 %. In addition, HPV 69, 71, 61, 26 and 33 were only detected in patients with multiple HPV types. The distribution rate (29 genotypes identified) of HPV isolates are mentioned in Figure 2. Single, multiple and un‐typeable HPV genotypes were diagnosed as follows: 1 type (318/655 [48.5%]), 2 types (162/655 [24.8%]), 3 types (83/655 [12.7%]), 4 types (42/655 [6.5%]), 5 types (21/655 [3.2%]), 6 types (8/655 [1.3%]), 7 types (4/655 [0.6%]), 8 types (1/655 [0.2%]) and un‐typeable 16/655 (2.4%) subjects, respectively (Table 2, Figure 1 and 2). From 655 HPV positive cases, 100 cervical scrapping specimens (15.3 %) were selected to be sequenced. Quality of PCR amplicons and HPV genotypes detected were the main inclusion and exclusion criteria for sequencing. However, 25 of failed sequenced amplicons were excluded and were not analyzed. The partial L1 gene of HPV genotypes of cervical scrapping specimens have been deposited in GenBank database under the accession numbers MH253467 to MH253566. The phylogenetic tree analysis showed the genetic variability among HPV genotypes detected in this study were closed to clusters from African, Asian, European and American isolates. The phylogenetic analysis of dominant common HR‐HPVs (HPV 16 and 18) and LR‐HPVs (HPV 6 and 11) were constructed separately (Figure 3, 4, 5, 6).

**Table 1:** Distribution of HPV subjects among different age groups

Age groups	Subjects No. (%)	Mean age average ± SD	HPV positive cases (%)
15–24	143 (12.6)	22.2 ± 1.8	106 (16.2)
25–34	614 (54.2)	29.6 ± 2.7	371 (56.6)
35–44	274 (24.2)	38.6 ± 2.8	138 (21.1)
45–54	85 (7.5)	48.2 ± 2.7	31 (4.7)
55–64	14 (1.2)	59.1 ± 2.8	8 (1.2)
≥65	3 (0.3)	67 ± 1.7	1 (0.2)
Total	1133 (100)	32.7 ± 8.0 (17–69 years)	655 (100)

Abbreviations: HPV, human papillomavirus; SD, standard deviation.

The highest prevalence rate of HPV genotypes detected in single and multiple infections in 655 positive cases are shown in Figure 1. The prevalence rate of the rest diagnosed genotypes was less than 3%. In addition, HPVs 69, 71, 61, 26, and 33 were only detected in patients with multiple HPV types.

The distribution rate (29 genotypes identified) of HPV isolates are mentioned in Figure 2. Single, multiple, and un‐typeable HPV genotypes were diagnosed as follows: 1 type (318/655 [48.5%]), 2 types (162/655 [24.8%]), 3 types (83/655 [12.7%]), 4 types (42/655 [6.5%]), 5 types (21/655 [3.2%]), 6 types (8/655 [1.3%]), 7 types (4/655 [0.6%]), 8 types (1/655 [0.2%]), and untypeable 16/655 (2.4%) subjects, respectively (Table 2, Figures 1 and 2). From 655 HPV‐positive cases, 100 cervical scrapping specimens (15.3%) were selected to be sequenced. Quality of PCR amplicons and HPV genotypes detected were the main inclusion and exclusion criteria for sequencing. However, the failed sequenced amplicons were excluded and are not shown in the report. The partial segments of HPV genotypes L1 gene have been deposited in the GenBank database under the accession numbers MH253467 to MH253566. The phylogenetic tree analysis showed the genetic variabilities among HPV genotypes detected in this study were close to clusters from African, Asian, European, and American isolates. The phylogenetic analysis of dominant common HR‐HPVs (HPVs 16 and 18) and LR‐HPVs (HPVs 6 and 11) were constructed separately (Figures 3, 4, 5, 6).

**Table 2:** Prevalence of HPV detected among population study

HPV genotypes	Percentage of detected and undetected %	Total of HPV detected and undetected cases (%)
1 Type	48.5	
2 Types	24.8	655 (57.8)
3 Types	12.7	
4 Types	6.5	
≥ 5 Types	5.3	
Un‐typeable	1.4	
HPV negative	42.2	478 (42.2)

Abbreviation: HPV, human papillomavirus.

## 4 DISCUSSION

HPV persistent infection is highlighted to be one of the most common causes of genital disorders throughout the world.^7^, ^11^ It would appear HPV genotypes are increasing in the normal population of low or unvaccinated communities. As a result, it can be a health warning for the incidence of cervical carcinoma and genital malignancies. It seems HPV infection is not restricted to a specific age group although the rate of HPV infections is more distinguished in subjects with unsafe sexual activities and multiple partners.^8^, ^10^, ^25^, ^30^, ^31^, ^32^, ^33^, ^34^, ^35^, ^36^, ^37^, ^38^, ^39^, ^40^, ^41^, ^42^ In the current cross‐sectional descriptive study, the highest prevalence rate of HPV genotypes (56.64%) was observed in the 25–34 years age group of 1, 133 cervical scrapping specimens. Our finding revealed that HPV genotypes detected were remarkable in the general population and multiple HPV genotypes were also observed as asymptomatic forms. The prevalence rate of HR and LR‐HPV genotypes were nearly similar to many scientific efforts; however, some evidence might indicate slight differences.^10^, ^23^, ^29^, ^31^, ^42^, ^43^ However, findings of HPV patterns and epidemiological prevalence rates vary in low or unvaccinated and fully vaccinated countries. Obviously, the percentage of specificity, sensitivity, and accuracy of molecular diagnostic methods play major roles in HPVs detection rate of clinical samples. Differences in HPV consequences are influenced by the most common effective factors such as the low amount of sample volume, sample collection‐shipment procedure, quality of LBC container/brush, inhibitors (due to taking some medications and topical ointments), and lab settings.^10^, ^22^, ^23^, ^24^, ^30^, ^43^, ^44^ Conclusively, HPV genotyping was performed using an approved CE marked and  IVD Kit (INNO‐LiPA® HPV Genotyping). The PCR amplicons of 100 subjects (15.3%) were amplified by My09/11 updated primers of the L1 major capsid region. Then, HPV‐positive isolates were determined and sequenced successfully. It seems the same common HR and LR‐HPV genotypes are cocirculated throughout Iran and other countries such as HR‐HPVs 16 and 18, and LR‐HPVs 6 and 11. However, some HPV genotypes, for example, HPV53 (probably HR), HPV31 (HR), HPV51 (HR), and HPV52 (HR) were also common in various ethnicities and continents. Overall, our findings were consistent with other scientific reports but a larger sample size of population study, genotyping with an approved diagnostic assay, and sequencing were advantages of the study.^29^, ^31^, ^34^, ^35^, ^45^, ^46^, ^47^, ^48^

The HPV genotypes lineages of phylogenetic outcomes revealed that detected types belonged to different genetic lineages from African, Asian, European, and American populations. Some types are supposed to have existed in specific regions although it seems traveling, tourism, and social communications increase the exposure risk of HPVs' different genotypes. Therefore, the various HPV genotypes were cocirculated between the communalities.^6^, ^8^, ^10^, ^19^, ^25^, ^45^, ^47^, ^48^, ^49^, ^50^, ^51^, ^52^, ^53^ Moreover, synergistic effects and trends in cocirculation of specific HPV genotypes are needed to be appraised in a future meta‐analysis study. Interaction and cocirculation of HR‐ and LR‐HPV genotypes in multiple infections might induce some mutations to generate new genotypes and maybe affect the severity of genital and anogenital infections.^9^, ^25^, ^26^, ^27^ However, there were no specific synergistic associations between genotypes in our last preliminary findings.^9^, ^25^ Furthermore, the remarkable rate of HPVs 44 (LR), 53 (pHR), 39 (HR), 51 (HR), and 66 (HR) detected in single and multiple infections suggested the nonavalent vaccines could be reformulated to boost immune responses.

## 5 CONCLUSION

To summarize, the distribution and burden of genital and anogenital malignancies are increasing through the multiple HPV genotypes in the normal population accordingly. Effective screening and diagnostic procedures besides enhanced vaccination strategy are needed to be implemented and extended in low or unvaccinated countries. The main outcome of the current survey addresses helpful insights into molecular epidemiologists and health bodies reports of HPV low‐vaccinated communities for designing and organizing the cancer care screening surveillance programs.

## AUTHOR CONTRIBUTIONS

Manijheh Vazifehdoost contributed to data analysis and prepared the draft of manuscript. Fatemeh Eskandari performed the experiments, collection, and sorting the specimens. Amir Sohrabi contributed to the conception, design, data interpretation, prepared, and revised the manuscript.

## CONFLICTS OF INTEREST

The authors declare no conflicts of interest.

## Data Availability

All data of study mentioned in the manuscript.
